# The role of IGF-pathway biomarkers in determining risks, screening, and prognosis in lung cancer

**DOI:** 10.18632/oncotarget.28202

**Published:** 2022-02-18

**Authors:** Alexander W. Pohlman, Hita Moudgalya, Lia Jordano, Gabriela C. Lobato, David Gerard, Michael J. Liptay, Christopher W. Seder, Jeffrey A. Borgia

**Affiliations:** ^1^Rush Medical College, Rush University Medical Center, Chicago, IL 60612, USA; ^2^Department of Anatomy and Cell Biology, Rush University Medical Center, Chicago, IL 60612, USA; ^3^Department of General Surgery, Rush University Medical Center, Chicago, IL 60612, USA; ^4^Department of Biochemistry, Rush University Medical Center, Chicago, IL 60612, USA; ^5^Department of Cardiovascular and Thoracic Surgery, Rush University Medical Center, Chicago, IL 60612, USA; ^6^Department of Pathology, Rush University Medical Center, Chicago, IL 60612, USA

**Keywords:** IGF, lung cancer, biomarkers, screening, prognostication

## Abstract

Background: Detection rates of early-stage lung cancer are traditionally low, which contributes to inconsistent treatment responses and high rates of annual cancer deaths. Currently, low-dose computed tomography (LDCT) screening produces a high false discovery rate. This limitation has prompted research to identify biomarkers to more clearly define eligible patients for LDCT screening, differentiate indeterminate pulmonary nodules, and select individualized cancer therapy. Biomarkers within the Insulin-like Growth Factor (IGF) family have come to the forefront of this research.

Main Body: Multiple biomarkers within the IGF family have been investigated, most notably IGF-I and IGF binding protein 3. However, newer studies seek to expand this search to other molecules within the IGF axis. Certain studies have demonstrated these biomarkers are useful when used in combination with lung cancer screening, but other findings were not as conclusive, possibly owing to measurement bias and non-standardized assay techniques. Research also has suggested IGF biomarkers may be beneficial in the prognostication and subsequent treatment via systemic therapy. Despite these advances, additional knowledge of complex regulatory mechanisms inherent to this system are necessary to more fully harness the potential clinical utility for diagnostic and therapeutic purposes.

Conclusions: The IGF system likely plays a role in multiple phases of lung cancer; however, there is a surplus of conflicting data, especially prior to development of the disease and during early stages of detection. IGF biomarkers may be valuable in the screening, prognosis, and treatment of lung cancer, though their exact application requires further study.

## INTRODUCTION

Lung cancer is the leading cause of cancer deaths in the United States, with an estimated 236,000 new diagnoses and 132,000 deaths expected in 2021 [1]. It is well established that lung cancers may present with a wide variety of phenotypic and mutational heterogeneity not only across the range of the disease, but also within specific histological subsets, such as lung adenocarcinoma. Yet, current modalities for screening and treating lung cancers employ broad guidelines that often do not account for the aforementioned molecular heterogeneity or tumor immune microenvironment, both of which may be of great importance for diagnostics and treatment plan formulation. Currently, screening for lung cancer is predicated almost exclusively on age and smoking history, while prognosis and treatment are dependent on the TNM (tumor, node, and metastasis) staging system. In more recent years, expansions to these treatment algorithms have developed as specific ‘driver mutations’ within cell-signaling pathways, such as EGFR, KRAS, and ALK, have been associated with ‘targeted’ therapeutic approaches. The pathophysiology of cancer, however, is known to be significantly more complex than even these systems acknowledge. The identification of numerous circulating biomarkers that attempt to provide further classification of these tumors promises the exploration of a new frontier in the screening and prognostication for a variety of cancers. The insulin-like growth factor (IGF) and other members of the IGF pathway, in this context, will be the point of this article.

### The IGF pathway

The IGF pathway is an intricate, multi-tiered dynamic of ligands, receptor types, and cell-signaling cascades with multiple levels of regulation. Broadly, IGF modulates cell behavior through endocrine, paracrine, and autocrine control [2]. Binding of IGF complexes to their respective receptors induces cellular adaptations that promote survival, proliferation, and invasion during normal human physiology and numerous types of cancer [3, 4].

Two insulin-like growth factors have been identified, IGF-I and IGF-II. Although IGF-II has been hypothesized to regulate fetal musculoskeletal cell differentiation and survival, and its molar ratio relative to IGF-I is 3:1 in adults, the understanding of the contours of its regulatory status is limited. Because adverse expression of IGF-II may impact a number of metabolic conditions, such as diabetes, postulations have suggested IGF-II continues to affect adipose and musculoskeletal tissue throughout life [5]. Reports also depict IGF-II involvement in phenotypic plasticity, potentially leading to more aggressive and resilient clones in progressive tumors [6, 7]. Exploration of IGF-II in this context is a highly-active and evolving research topic.

In contrast, extensive documentation of IGF-I’s function has revealed an association between dysregulation of IGF-I and tumorigenesis. Canonically, it is well established that growth hormone (GH) stimulates liver production and release of IGF-I, which subsequently exerts endocrine-related functions. Individuals with acromegaly and abnormally elevated levels of GH also possess a concomitant augmentation of IGF-I levels, and have been observed to have increased incidence of multiple types of malignancies [8, 9]. These endocrine features contrast with the autocrine and paracrine characteristics of IGF-1 that originate within the tumor. Lung cancer tissue contains differential expression of multiple molecules within the IGF axis, including the boosted production of IGF-I, IGF-II, and IGF-1 receptor (IGF-1R) and decreased expression of IGF binding protein-3 (IGFBP-3). Modulated expression of these molecules is well-documented to be associated with aggressive disease and poor clinical outcomes [10].

**Figure 1:**
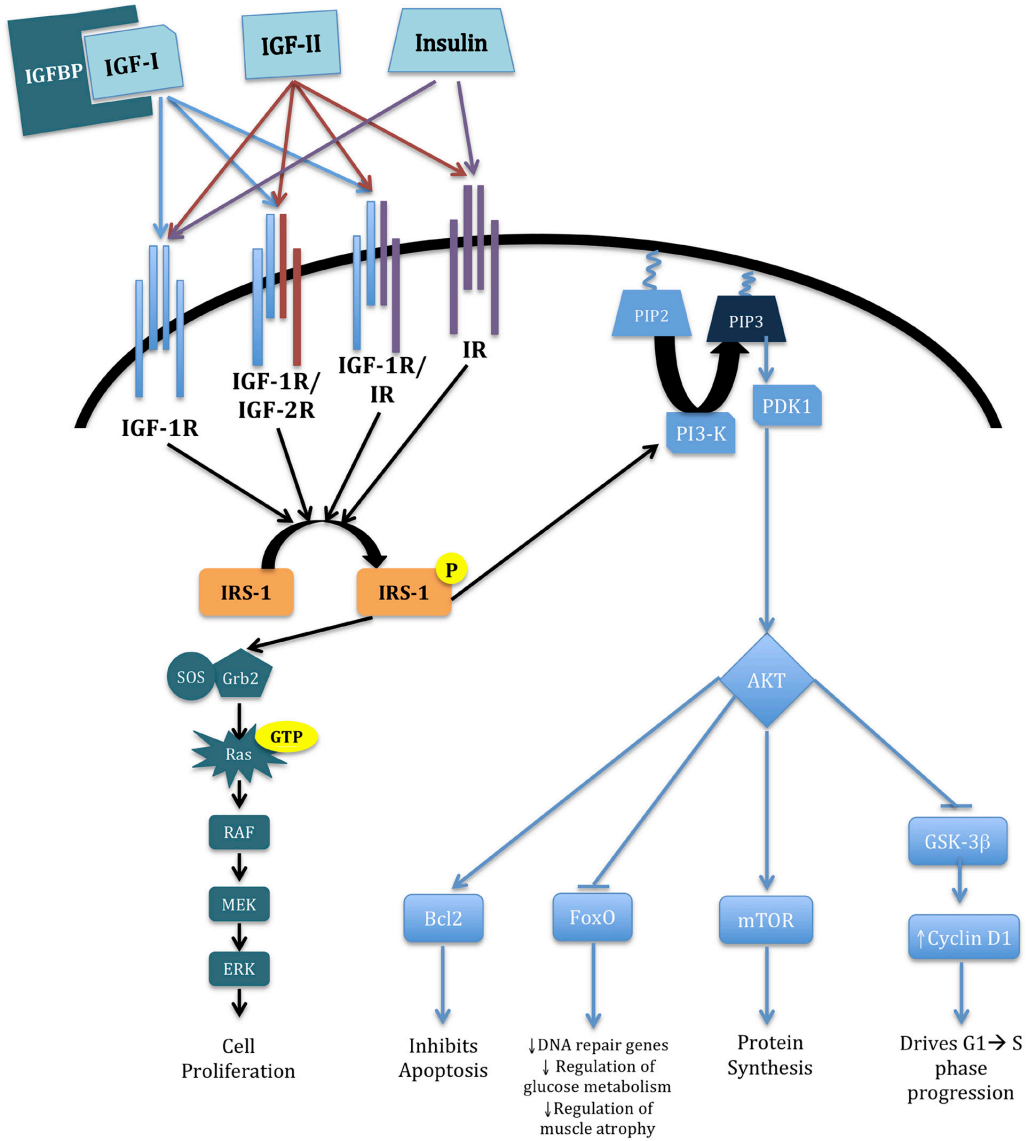
IGF cascade. Broad overview of the IGF pathway and its downstream effects on cell survival and proliferation. Briefly, binding of IGF-I to IGF-1R begins the cascade via two separate pathways via phosphorylation of IRS-1. The K-Ras/BRAF/MEK/MAPK pathway increases cell proliferation. The PI3-K/AKT pathway affects several downstream regulators that have varying effects within the cell. Stimulation of Bcl2 inhibits apoptosis; inhibition of FoxO diminishes DNA repair, glucose metabolism, and regulation of muscle atrophy; activation of mTOR promotes protein synthesis; and abrogation of GSK-3β increases Cyclin D1 levels, resulting in phase progression in the cell cycle [12, 19–21].

The mechanism by which IGF navigates its numerous effects is via multiple downstream signaling events following ligand-receptor binding. IGF-I signaling is mediated through several potential receptor complexes, including IGF-1R homodimers or IGF-1R/IGF-2R or IGF-1R/insulin receptor (IR) heterodimers [11]. Binding of IGF-I to its constitutive receptor complex activates insulin receptor substrate-1 (IRS-1), which instigates signaling through the Akt and K-Ras/BRAF/MEK/MAPK pathways. The Akt pathway encourages decreased apoptosis increased protein synthesis, and augmented glucose metabolism via B Cell Lymphoma-2 (Bcl2), mTOR, and GSK-3β, respectively. The K-Ras/BRAF/MEK/MAPK cascade promotes cell proliferation [12]. IGF-II supplements the complexity of this environment because of its ability to direct identical signaling cascades. IGF-II normally binds IGF-2R, but when IGF-2R activity is compromised, it may bind the IGF-1R/IGF-2R heterodimer or IR thus causing the same downstream effects [11, 13, 14]. These aforementioned critical alterations in cell metabolism and proliferation can confer cancer cell survival. Correlations between IGF cell-signaling and malignancies, including breast, ovarian, prostate, and colon, are based on up-regulated/down-regulated levels of pathway markers in association with cancer development and growth [15–18]. These canonical routes for IGF-1 modulated signaling are illustrated in Figure 1.

The IGF pathway has also been implicated in lung tumorigenesis. First noted in 1986, malignant lung tissue contained a higher concentration of IGF-I than normal lung tissue from the same patient [22]. More recently, it was discovered serum IGF-I levels diminished post-operatively after the resection of lung cancers. This decrease was most pronounced with the removal of tumors larger than 3 cm, suggesting the potential magnitude of tumoral IGF-1 production [23].

In circulation, IGF-I is most commonly sequestered within a ternary complex with one of the insulin-like growth factor binding proteins (IGFBPs) along with an insulin-like growth factor acid-labile subunit (IGFALS). However, IGF-I can also exist as an unbound ‘free’ form or as a binary complex with IGFBP. IGFBP’s have a much greater affinity (close to one order of magnitude) for IGF-I as compared to its receptor [24]. Six structurally related high-affinity binding proteins (IGFBPs 1-6) and additional low-affinity binding proteins, such as IGFBP-7, continue to be isolated. The low-affinity binding proteins demonstrate less genetic and structural conservation when compared to IGFBPs 1–6 [25, 26]. The IGFBPs generally inhibit the actions of IGF by sequestering it from IGF-1R, however, rendering this complex as limiting IGF-I bioavailability may be an oversimplification. IGFBPs also modulate the activity of IGF at the receptor, thereby extending its half-life in circulation, controlling its egress from the vasculature, and influencing its clearance [24, 27, 28]. IGFBPs likely also independently regulate receptor activation and downstream signaling. For example, IGFBP-3 blocks IGF-1R stimulation irrespective of IGF-I binding. These effects are precipitated based on specific IGFBP and receptors. For example, IGFBP-3 does not independently affect IR, and other IGFBPs, such as IGFBP-1 and IGFBP-5, have no independent effects on IGF-1R [28].

Not surprisingly, the different binding proteins have unique properties and functions. IGFBP-3 is the most abundant IGFBP in the blood and operates as the transport workhorse for the IGF protein superfamily, ordinarily carrying approximately ninety percent of circulating IGF. Consequently, its main IGF-dependent function is to control the amount of free IGF in circulation, which furnishes numerous downstream effects, including the proportioning of IGF for cellular proliferation or the enhancement of apoptosis. IGFBP-3 also has IGF-independent functions such as interacting directly with cell surface receptors, attenuating cell surface receptor affinity for IGF, thereby allowing the growth factor to bind to other cells. Intracellular targets, such as retinoid acid X receptor alpha (RXR-α), permit IGFBP-3 to control gene expression. The overall role of IGFBP-3, therefore, is somewhat unclear due to the complex mechanisms of action that rely on the surrounding milieu, including availability of cell surface receptors, internal cell targets, and IGF levels [13, 24].

Although IGFBP-3 is the cardinal regulator of IGF-I activity and, by extension, has a highly significant impact on tumorigenesis, the other IGFBPs have important biological roles in the inhibition or potentiation of IGF, as well [29–31]. The roles of many of these binding proteins, however, are still incompletely described, and their significance as possible markers for lung cancer are not as well-documented as IGFBP-3. A brief summary of the other main IGFBPs follows, but it is important to note a full discussion of their functions in different cancers and normal physiology is beyond the scope of this review.

IGFBP-1 abrogates DNA synthesis, cell growth and differentiation yet also amplifies IGF-I action when combined with certain reagents, such as platelet-poor plasma, or in certain cell lines (e.g., MDA-231 breast carcinoma cells). IGFBP-2 is dichotomous in nature, as it both weakly enhances and prevents IGF activity; it is primarily present in the nervous system. IGFBP-4 is primarily an IGF inhibitor in most environments. IGFBP-5 proscribes most IGF-I actions, except when surrounded by fibroblast extracellular matrix, a surrounding that inverses its abilities by strengthening the effect of IGF-I [32]. IGFBP-5 usually sequesters IGF to the intravascular space when in its ternary complex, but the binary IGFBP-5/IGF-I transits to the extracellular space [33]. As with the other IGFBPs, IGFBP-6 imparts inhibitory effects on IGF and mostly manages gonadotropic activity. It is important to note different tissue and cellular landscapes and post-translational modifications, such as phosphorylation, substantially govern the correlation between the IGFBPs and IGF, manifesting currently enigmatic relationships [33]. Refer to Table 1 for a summary of these functions.

**Table 1:** IGFBP functions

IGFBP	Function	Major sites of expression
**1**	Mostly inhibits DNA synthesis, cell growth, and differentiation. Potentiates IGF-1 action when combined with platelet-poor plasma or certain cancer cells.	Liver, placenta, and endometrium
**2**	Weakly potentiates and inhibits IGF activity.	Liver, pancreas, nervous system
**3**	Transports 90% of IGF in circulation. May sequester IGF, thus causing apoptosis. May also directly bind cell surface receptors, causing altered gene expression and altered affinity for IGF cell receptors. Functions change due to the surrounding environment (i.e., IGF levels or available cell receptors and targets)	Placenta; notably, it is found in large quantities in circulation.
**4**	Mostly inhibits IGF as well as growth of many cancers (i.e., colon cancer); donor in presence of PAPP-A	Widely expressed throughout the body, especially in ovary and liver.
**5**	Has inhibitory, stimulatory, and independent effects throughout the body, especially in the musculoskeletal system.	Testis, ovary, trabecular meshwork, bone, lung, uterus, placenta.
**6**	Mostly inhibits IGF-II and cancer growth.	Highest expression is in gonadal/reproductive tissue.

Overview of known IGFBP functions and their major sites of expression [34–37].

These interactions are further complicated by the presence of proteases. Proteolytic cleavage of binding proteins can release free IGF, free fragments of binding proteins, or destabilize the affinity of the binding protein for its ligand, propagating the binding of IGF to the cellular receptors. Such enzymes belong to the various classes of cell-surface proteases, including serine proteases, cathepsins, matrix metalloproteinases, and PAPP-A, and are specific for the different IGFBPs [33]. Some binding proteins also develop IGF-independent actions after being post-translationally modified via proteolysis. In particular, post-proteolytic IGFBP-3 protein fragments are capable of IGF-independent activation of IGF-1R [18].

Within the tumor immune microenvironment (TME) of various cancers, these inter-balances gain an even greater intricacy. IGFs have classically been cast as systemic modulators, but research has introduced these growth factors as likely paracrine and cytokine-like actors within TMEs. Such a distribution of activity is of particular importance due to the abundant expression of IGF-1Rs of certain immune cells, such as monocytes and CD4+ T-helper cells. IGF-I’s anti-apoptotic effects on these immune cells may have a great impact on tumor survival. Whether IGF alters the TME into an anti-tumor environment or a protective environment for the tumor remains to be clearly demonstrated [38].

The interplay between IGF, its binding proteins, the resultant effects on IGF function, and the contributions of other members of the cellular environment constitute a complex system. It is this dynamic that lends great depth, difficulty, and promise to the study of the IGF system in cancer.

### IGF biomarkers and risk of developing lung cancer

Delineating the risk of lung cancer development is the most important factor for the prevention and screening of the disease. Currently, the primary means of lung cancer prevention is accomplished via smoking abstention or cessation and is further supplemented by early diagnosis via low-dose CT (LDCT) radiography to help reduce mortality [39]. However, LDCT scans are largely restricted to those with broad risk factors for development of disease, including age and smoking history. Due to these relatively simplified metrics, high numbers of false positive results are recorded (the false positive rate per screening round was 23.3% in the original National Lung Screening Trial (NLST)), leading to the profligate consumption of resources, expansion of healthcare costs, and prescription of unnecessary invasive procedures (1.8% of NLST participants with a false positive result) [39]. There is a need, therefore, for the designation of more specific risk factors that may predict the future development of lung cancer. One possibility is the use of biomarkers, such as those found within the IGF pathway. The current evidence for the selection of members of the IGF pathway as viable signposts for lung cancer diagnoses is unclear, and the lack of published reports specifically designed to measure IGF pathway family member levels prior to the diagnosis of disease presents an obstacle. In this section, only evaluations of blood samples acquired prior to diagnosis of disease, which thus assessed the actual risk of development of the cancer rather than the detection of an existing cancer, will be discussed. Establishing such experimental design parameters, unfortunately, limits the available pertinent data in the literature for a true meta-analysis, which is further complicated by conflicting results.

One prospective case-control analysis of 1695 ever smoker patients found a statistically significant, inverse association between IGF-I and the development of lung cancer (HR = 0.91; 95% CI, 0.86–0.96) [40]. Multiple other presentations, however, reported no statistically significant correlation between IGF-I levels prior to diagnosis and the onset of the disease [41–44]. Two of these studies, each with 159 and 1143 case subjects, demonstrated an elevated risk of lung cancer development with increased IGF-I levels, but the results were not statistically significant [42, 45]. An inverse relationship between IGF-I levels and lung cancer development was described in a different paper with 200 case subjects, but this aspect ceased to be statistically significant after accounting for body mass index (BMI) and smoking history [43]. As such, no definitive relationship between IGF-I levels and the development of lung cancer has been proposed. Also, the non-statistically significant nature of apparent associations upon the inclusion of additional criteria, such as BMI or smoking history, signals a host of external factors likely influence IGF-I concentration prior to disease occurrence and contribute to its variable and complex expression pattern.

Five of the six previously mentioned articles also checked IGFBP-3 levels. Similar to IGF-I, no consensus was maintained among the accounts concerning how IGFBP-3 affects the development of lung cancer. An inverse relationship between IGFBP-3 and lung cancer in ever-smokers was offered in two papers, whereas another investigation revealed augmented IGFBP-3 correlated with advancement of lung cancer [41, 42, 45]. The remaining two studies demonstrated no statistically significant tendency between IGFBP-3 and initiation of lung cancer [43, 44]. One of these trials also measured IGFBP-1 and IGFBP-2 levels, which were concluded to not be significantly related with development of lung cancer in women [44]. For reference, Table 2 summarizes several of these finding.

**Table 2:** Results of studies on risk of development of lung cancer

Author	Year	Design	Cases	Controls	Time from Sample to Diagnosis	IGF-I vs. Risk of Lung Cancer	IGFBP-3 vs. Risk of Lung Cancer
Qian, et al. [40]	2020	Prospective Cohort	1695 ever smokers; 301 never smokers		0+ years	Inverse association^b^	–
London, et al. [41]	2002	Prospective Case-Control	230	659	0+ and 2+ years	NS	Inverse association^b^
Spitz, et al. [42]	2002	Nested Case-Control	159	297	3+ years	Inverse association^a^	Highest quartile had increased risk
Ahn, et al. [43]	2006	Nested Case-Control	200	400	5+ years	NS^c^	NS^c^
Lukanova, et al. [44]	2001	Nested Case-Control	93	186	6+ months and 3+ years	NS	NS
Ho, et al. [45]	2016	Nested Case-Control	1143 ever smokers	1143	1 year	NS	Inverse association

Abbreviation: NS: Not significant. ^a^Reported result of those below the age of 60 when controlling for IGFBP-3 levels; all other stratifications were not statistically significant. ^b^Result seen only in ever smokers; ^c^Significant difference seen until adjustment for BMI and smoking history.

Another meta-analysis of 2686 lung cancer patients examined common polymorphisms within the IGF axis, finding that certain patients had a predisposition to lung cancer due to genetic variations in IGF-I, IGF-II, IGF-1R, IGFBP-3, and IGFBP-5 [46]. However, on subgroup analysis, the study found that this outcome was only present in the Asian population, population-based studies, hospital based studies, and PCR-RFLP (restriction fragment length polymorphism) studies, and it was not present in the Caucasian population. Therefore, this study further points towards the complexities of the IGF system prior to the development of cancer including how patient demographics and genetic make-up may influence it.

While additional research may provide greater clarity and perspective, current evidence intimates IGF markers are not beneficial in the determination of lung cancer risk, possibly due to the cross-talk of the IGF signaling pathways with other cascade highways, the impact of environmental, lifestyle, and genetic factors, or unknown stimulatory/inhibitory agents prior to the development of lung cancer.

### IGF biomarkers and lung cancer screening

The publications of the NLST results still prompted the National Comprehensive Cancer Network to recommend the administration of LDCT as the preferred screening application for the detection of lung cancer for appropriately selected high-risk patients [39, 47]. Due to the high false positive rate, the International Association for the Study of Lung Cancer (IASLC) and the Strategic CT Screening Advisory Committee (SSAC) advised the utilization of blood-based biomarkers to assist current LDCT screening [48]. Multiple efforts to establish a “liquid biopsy” capable of reducing false-positives screens, prognosticating risk of developing cancer, and navigating patient care have been initiated. Of the potential biomarkers identified, candidates within the IGF pathway have emerged as contenders. However, the literature is replete with non-standardized techniques and conflicting results, causing difficulty in formulating definitive conclusions at this time.

One of the largest issues with the currently available data involves the combination of trials that isolated serum and plasma with different protocols and the incomplete description of the methodology (duration of sample storage prior to centrifugation; types of reagents that dissociated the IGF from its binding partner; the possible application of IGF-II to prevent re-association; etc.), which had a significant impact on analytical results. That is, pre-analytical variables in the matrix of choice have been shown to alter IGF-I level measurement [49]. Although serum and plasma are similar in composition, plasma includes the soluble proteins responsible for blood clotting, whereas blood that has undergone the myriad of proteolytic steps that constitute the clotting cascade forms serum. Additionally, specific details of procedures and types of anti-coagulants may vary, possibly influencing which metabolites may remain in the processed sample. One study indicated data point reproducibility is high within the same procedure, but serum tends to contain higher metabolic concentrations than plasma and is thus more sensitive for biomarker analysis [50]. A separate paper specifically showed the effect elicited by different isolation procedures: Samples were either treated with an acid extraction solution that induced IGF-IGFBP complex dissociation as a method to detect total IGF-I in the blood, serum, or plasma or remained untreated. The unextracted (non-dissociated) serum contained markedly elevated IGF-I when compared to controls, while the extracted (dissociated) serum did not, suggesting a significant source of potential measurement bias [51]. Such a fundamental difference either between serum and plasma or the protocols applied to them may account for some of the variance between studies and create complications in the comparison or combination of current data sets. Current practice makes unextracted serum inappropriate for IGF measurement, but much of the current data was gathered prior to this normalization.

In 2011, the first international consensus statement on the measurement of IGF was released [52, 53]. The consortium encouraged the uniform use of the IS 02/254 WHO reference standard for IGF assays, the choice of serum for test samples, a delay of no more than two hours from blood acquisition to centrifugation, the commitment to a validated method for preventing IGFBP interference, and the consideration of multiple IGF measurements due to intra-individual imprecision. Therefore, as more studies are performed with consistent adherence to these guidelines, it is possible less discordance will exist among the data, and a more clearly defined role for IGF biomarkers in lung cancer screening will develop.

Of the existing data, multiple reports have discovered elevated serum or plasma IGF-I concentrations in patients with existing primary lung cancers [54–59]. Four investigations were cross-sectional, and two prospective cohort studies totaled approximately 500 case subjects. Participant serum or plasma was analyzed via enzyme-linked immunosorbent assay (ELISA), radioimmunoassay (RIA), or immunoradiometric assay (IRMA), with the majority of the trials analyzing serum samples via ELISA. Most inquiries encompassed non-small cell lung cancer (NSCLC), while one investigation also included small cell lung cancer (SCLC) [55]. The key message from these interrogations was the apparent elevations in IGF-I levels in relation to tumor size, advanced tumor stage, and metastatic propensity. A statistically significant difference between histological subtypes of lung cancer was not revealed.

Trials that concerned IGFBP-3 typically described lower levels of the binding protein in all lung cancers. Also, heightened differences were observed between IGF-I and IGFBP-3 levels in control participants compared to enrollees who had a higher T stage; revealed cancer of the lymph nodes; and demonstrated evidence of metastases [57–60]. A synopsis of the results stipulates IGF-I generally increases with lung cancer, especially individuals diagnosed with advanced disease, while IGFBP-3 decreases. This phenomenon may be a consequence of the ability of IGFBP-3 to bind IGF-I, thereby suppressing its proliferative and anti-apoptotic functions. Therefore, a coinciding reduction of IGFBP-3 and elevation of IGF-I may permit increased tumor growth to occur. Although the cause-effect dynamic of these two potential biomarkers and the instigation of cancer is still not well-established, the cited studies seemingly suggest the future employment of these biomarkers for screening in lung cancer.

This relationship is not as obvious, however, as the above citations may surmise. Other publications contradict the previously mentioned generalization with reports of lower concentrations of IGF-I in the serum of lung cancer patients [51, 60]. Notably, one of these counterposing papers included a much larger patient sample size (224 case subjects and 123 controls) than the encounters listed above, indicating a greater statistical power [33]. This manuscript, similarly, demonstrated highly significant (*p* < 0.001) lower circulating levels of IGF-II, IGFBP-3 and IGFBP-5 in the plasma for screening cases with malignancies versus those with benign pulmonary nodules. Further and more intensive analyses, therefore, are necessary to dissect any relationship or concentration-dependent conjunction of these putative members of the IGF family. A 2012 meta-analysis examined the data from six nested case-control groups and eight case-control studies totaling 401 case subjects to discern the association between IGF-I and IGFBP-3 levels and the presence of lung cancer. No statistically significant correlation between IGF-I levels and the presence of lung cancer was detected. The analysis did, however, indicate a statistically significant, inverse relationship between IGFBP-3 levels and the existence of lung cancer [61]. Although a reconciliation of the discrepancy presented in prior publications for IGF-I levels was not achieved, the consideration of IGFBP-3 as a potential biomarker for lung cancer was further supported. Table 3 lists a brief summary of major papers on this topic.

**Table 3:** Results of papers studying detection of lung cancer

Author	Year	Design	Cases	Controls	Sample	Method	IGF-I Status	IGFBP-3 Status
Reeve, et al. [51]	1990	Cross-Sectional	52	63	Serum	RIA, IRMA	↓	–
Fu, et al. [54]	2013	Prospective Cohort	80	45	Serum	ELISA	↑	–
Izycki, et al. [55]	2004	Prospective Cohort	38	10	Serum	ELISA	↑	–
Tisi, et al. [56]	1991	Cross-Sectional/Cohort	46	38	Serum	RIA	↑	–
Wang, et al. [57]	2004	Cross-Sectional	78	14	Serum	RIA, IRMA	↑	NS
Wang, et al. [58]	2013	Cross-sectional	57	17	Serum	ELISA	↑	↓
Yu, et al. [59]	1999	Cross-Sectional	204	218	Plasma	ELISA	↑	↓^d^
Kubasiak, et al. [60]	2014	Cross-Sectional	224	123	Mixed (serum/plasma)	Luminex	↓	↓
Cao, et al. [61]	2012	Meta-Analysis	401	343	Mixed (mostly Serum)	RIA, ELISA, IRMA	NS	↓

Abbreviations: NS: Not significant; RIA: Radioimmunoassay; ELISA: Enzyme-linked immunosorbent assay; IRMA: Immunoradiometric assay. ↑ Higher levels seen in lung cancer. ↓ Lower levels seen in lung cancer. ^d^Result after adjustment for IGF-I level.

Biomarker research in recent years has shifted towards the use of IGFBPs outside of IGFBP-3, which may potentially widen the array of biomarkers within the IGF system for lung cancer detection. A 2021 study of 60 lung cancer patients found higher levels of IGFBP-4 in all stages of disease and histologic subgroups of lung cancer when compared to healthy individuals. It is also important to note that pregnancy-associated plasma protein A (PAPP-A) has proteolytic activity on IGFBP-4, so the study also measured these levels. Although PAPP-A levels did appear to be higher in untreated lung cancer patients when compared to healthy controls, these results were not statistically significant [62]. Additionally, IGFBP-2 has been studied in association with anti-IGFBP-2 autoantibodies in lung cancer. Notably, the highest sensitivity (85.7%) of these biomarkers for the diagnosis of lung cancer was seen when the autoantibodies and IGFBP-2 were used in combination [63]. A 2014 study of 224 case subjects measured levels of IGF-I, IGF-II, and IGFBPs 1-7 and found that IGFBP-5 and IGF-II levels were higher in benign tumors than in NSCLC [60]. Based on these recent studies, it is clear that the IGF system is full of potential biomarkers that warrant further study outside of the previously mentioned IGF-I and IGFBP-3.

Due to the complexity of the IGF system, the complexities and indeterminate nature of the tumor immune microenvironment, and the intricate interplay between the two, a few laboratories have attempted to manufacture panels of biomarkers that can better detect lung cancer. One group obtained 122 samples from patients with NSCLC and compared them to 225 healthy control samples [64]. Thirty previously tested analytes that demonstrated promise as biomarkers were determined via the random forest method. The top five ranked biomarkers, IGF-I, A1AT, CYFRA 21-1, RANTES, and AFP, were incorporated into a multi-analyte panel. This collection was then applied to a validation cohort of 21 NSCLC patients and 28 healthy control patients, in which it distinguished NSCLC patients from control patients at approximately 90% accuracy [64]. Another team specifically investigated the difference in levels of IGF-I, IGF-II, and IGFBP 1-7 between patients diagnosed with NSCLC (*n* = 224) and participants with benign pulmonary nodules (*n* = 123), as discovered on low-dose CT scans [60]. Analysis of differences in the IGF pathway biomarkers of the two cohorts spurred the application of samples into a multi-analyte kit constituting IGFBP-4, IGFBP-5, IGF-II, interleukin-6, interleukin-10, interleukin-1 receptor antagonist, and the stromal cell-derived factor-1 (SDF-1α+β). This test produced a negative predictive value of 100% on validation [60]. These studies add credence to the idea that increased usage of IGF pathway biomarkers may increase the utility of biomarker panels in lung cancer screening. However, additional and larger studies will be needed to corroborate these findings and to solidify the predictive capabilities of their levels.

### IGF biomarkers and prognosis in lung cancer

In addition to screening, a number of possible members of the IGF pathway have been postulated as having potential prognostic or predictive value pertaining to disease progression or treatment efficacy. Although IGF-I and IGFBP-3 have been emphasized regarding the categories of lung cancer risk and associated screening, additional biomarkers emerge during the course of the disease that may also accurately convey such an appraisal, including other IGFBPs, insulin receptor substrate (IRS)-1, IRS-2, and IGF-1R.

The reduction of the IGF-I/IGFBP-3 ratio in NSCLC patients who responded to first-line treatment suggested such a metric could be a valuable predictor of response to chemotherapy in these patients [65]. The association of high IGF-I levels with advanced stage disease, larger tumor diameter, and shorter survival was also indicative of these characteristics. Additionally, patient IGF-I levels were depressed following resection of NSCLC tumors, further demonstrating IGF-I as a prognostic biomarker that could be measured throughout the course of disease [23]. Increased IGFBP-3 levels prior to treatment with irinotecan and cisplatin chemotherapy were affiliated with improved prognoses in NSCLC patients with advanced disease, implicating a potential role of IGFBP-3 as a predictive biomarker [66].

The mediation of the expression of signaling components by IGF-I may be related to phenotypic transdifferentiation of the cancer cells via the epithelial-to-mesenchymal transition (EMT) spectrum, whereby tumor cells tend to lose adhesion to surrounding cells, thus increasing motility, invasion and metastasis of epithelial tumors [67, 68]. The elevation of IGF-I and IGF-1R and their resultant interaction appears to up-regulate the PI3K/AKT/NF-κB pathway with the concomitant activation of ZEB2 and SNAIL1, altering protein expression and the EMT phenotype in certain lung cancers [67]. The extended interplay of stimulatory and inhibitory checkpoints of the intracellular avenues of the IGF pathway is certainly more heavily regulated and interspersed with cross-pathway entrance ramps than such simplified explanations imply, but a full discussion of this pathway and the differences between cancer types is beyond the scope of this review. The general concept persists, however, of a paradigm in which IGF-I may directly affect the expression of other members of the IGF system, which may subsequently be used to determine overall prognosis and response to future treatments.

Additional potential candidates that may develop during the course of disease include IGF-1R, circ-IGF-1R (a form of circular RNA that affects gene expression at transcriptional or post-transcriptional levels), IRS-1, and IRS-2. Multiple meta-analyses of NSCLC patients correlated augmented expression of IGF-1R with worse disease-free survival (DFS) [69, 70]. No description, however, was delineated between overall survival (OS) and IGF-1R levels in NSCLC and SCLC. It is speculated inconsistencies in IGF-1R measurement techniques and variance of treatment between patients may have impacted the lack of findings in relation to OS [69]. Additionally, one study noted an inverse association of circ-IGF-1R with tumor size and lymph node metastasis, which may also be used as a related biomarker to signal worse prognosis [71]. Another study highlighted the coincidence of IRS-1 suppression and IRS-2 elevation, both significant substrates of IGF-1R, which was associated with worse outcomes in NSCLC [72].

As previously mentioned, most IGFBPs exhibit some utility in prognostication as well. One study linked IGFBP-1 to poor OS in lung adenocarcinoma [73]. IGFBP-1 was evaluated in a seven-analyte panel to identify patients with disease recurrence following resection of node-negative NSCLC tumors that were less than 4 cm in size. The panel proved to be 91% sensitive and had a negative predictive value of 83% [74]. Multiple papers have demonstrated that higher levels of IGFBP-2 are associated with worse OS in lung adenocarcinoma, squamous cell carcinoma, and small cell carcinoma, and these higher levels are associated with increased rates of metastasis and higher staging [73, 75, 76]. However, one of these studies did associate high IGFBP-2 levels with favorable OS in patients with squamous cell carcinoma [73]. Results for IGFBP-4 have shown some discrepancy between *in vivo* and *in vitro* studies. Two *in vivo* studies indicated poor prognostic associations with IGFBP-4, including worse OS and shorter median survival [73, 77]. *In vitro* studies have shown anti-tumor effects of IGFBP-4 in NSCLC [78]. Multiple studies showed an inverse association between IGFBP-5 and prognostic indicators, including OS in patients with lung squamous cell carcinoma, nodal status, and disease recurrence, and recurrence-free survival [73, 79]. High IGFBP-7 was also associated with spread to regional lymph nodes, but was dissociative with respect to recurrence-free survival [79, 80]. Finally, IGF2BP3 has shown an association with poor OS in lung adenocarcinoma [81, 82]. Though previous investigations insinuated some viability of IGFBPs in the prognostication of lung cancer, expanded clarity and a more extensive mapping of which IGFBPs are the most effective and potent markers for the prognostication of different types of lung cancer still remains to be ascertained. Future cases must concentrate on the concatenation of prognoses with regard to treatment strategies.

The ability of IGF biomarkers to serve as chaperones to the response to specific therapies has been proposed. For example, IGF-independent effects have also been observed in lung cancer resistance. IGFBP-2 appears to stimulate growth and is aligned with NSCLC resistance to dasatinib, a tyrosine kinase inhibitor (TKI), a group of drugs that interfere with tyrosine kinases, enzymes responsible for the propagation of cell signaling pathway activation [83]. Decreased IGFBP-3 in the peritumoral environment in NSCLC establishes a resistance to EGFR-TKIs, such as gefitinib and erlotinib, as well as cisplatin-resistant tumors. Also, diminished IGFBP-7 imbues NSCLC tumors with apparent cisplatin-resistant attributes [84–86]. Such evidence supports the notion that the presence or absence of IGFBPs may predict tumor response to selected therapies.

IGF-1R is also predictive of response to treatment, as demonstrated by the apparent up-regulation in IGF-1R in patients with NSCLC who have developed a resistance to gefitinib, an epidermal growth factor tyrosine kinase inhibitor (EGFR-TKI) [87]. It has been postulated this activation of the IGF system is a reactive compensatory mechanism due to the inhibition of EGFR by gefitinib. The exact nature of the cross-talk between these classic signaling cascades is likely a more complex interplay that is further confounded by the participation of the underlying tumor immune microenvironment. Also, the aforementioned IGF-1R-induced EMT may instigate resistance to erlotinib, another EGFR-TKI [88]. The accumulation of the data thus posits IGF-1R levels may help predict treatment response to a number of EGFR-TKIs.

The action of the putative inhibitor of IGF-1R as it relates to chemotherapeutic responsiveness has also been elaborated. The inhibitory hindrance of IGF-1R allowed gefitinib to reclaim some of its apoptotic and anti-proliferative properties in gefitinib-resistant NSCLC cell lines [89]. Similar findings relevant to circulating members of the IGF axis were also noted in the literature [6]. More recent human trials of a number of different IGF-1R inhibitors, however, display conflicting results. It is important to note that more than ten IGF-1R inhibitors, with varying structures/mechanisms, including TKIs and monoclonal antibodies, have been applied in clinical trials. The combination of these inhibitory factors with different chemotherapy agents has sparked varying degrees of success. Most of these trials did not reveal great efficacy in the treatment of lung cancers; however, patients typically were not selected based on specific biomarker levels [90]. For example, a cohort that combined Figitumumab, a monoclonal antibody targeting IGF-1R, with paclitaxel and carboplatin to combat advanced NSCLC generated greater progression-free survival in patients with squamous cell carcinoma during phase 2 trials, but increased deaths of subjects in phase 3 trials. The division of patient groups by the level of expressed IGF-I yielded two distinct groups: Patients with higher IGF-I levels had better outcomes and OS relative to the control group, while participants with low IGF-I levels showed worse OS compared to the control group [91]. A predictive pattern regarding IGF-I-associated response to treatment was therefore pronounced, and the corresponding selection of the proper patient populations for use, as well as an identification of a contraindication may be applicable in certain patients. A summary of studies on IGF system biomarkers and prognosis in lung cancer can be seen in Table 4.

**Table 4:** Papers studying prognosis in lung cancer

Author	Year	Study design	Sample size^*^	Sample	Method
Kotsantis, et al. [65]	2019	Prospective Cohort	73	Plasma	ELISA
Han, et al. [66]	2006	Prospective Cohort	77	Plasma	ELISA
Xu, et al. [69]	2019	Meta-analysis	3859	Tissue	IHC, RT-PCR
Zhao, et al. [70]	2014	Meta-analysis	3294	Tissue	IHC, RT-PCR, FISH
Piper, et al. [72]	2019	Retrospective Cohort	107	Tissue	IHC
Seder, et al. [74]	2017	Retrospective Cohort	123	Serum	Luminex FlexMAP 3D system
Guo, et al. [75]	2013	Case-control	164	Plasma	ELISA
Hu, et al. [76]	2014	Retrospective Cohort	110	Tissue	IHC
Shersher, et al. [79]	2011	Prospective Cohort	100	Serum	Luminex 100 IS System
Chen, et al. [80]	2020	Retrospective Cohort	60	Serum	ELISA
Shi, et al. [81]	2017	Retrospective Cohort	809	Tissue	RNA-seq
Guo, et al. [82]	2021	Retrospective Cohort	415	Tissue	RNA-seq
Langer, et al. [91]	2014	Randomized Controlled Trial	681	Serum	ICMA

Abbreviations: NS: Not significant; ELISA: Enzyme-linked immunosorbent assay; IHC: Immunohistochemistry; RT-PCR: Reverse Transcription polymerase chain reaction; FISH: Fluorescence *in situ* hybridization; ICMA: Immunochemiluminometric assay. ^*^Includes only cases of cancer, not controls.

Although the fluid cause-effect ecosystem between IGF signaling cascades and chemotherapy resistance glides between cresting and crashing waves of stimulation and inhibition that seem to simultaneously augment and cancel each other, many of these biomarkers may be clinically practical for the prediction of the response in targeted or individualized therapies.

## CONCLUSIONS

The role of the IGF pathway in the development, recurrence, or defeat of lung cancer, and its corresponding use in prediction, detection, and prognostication of disease is at the nexus of complex signaling cascades, numerous external factors, and a host of genomic, proteomic, and metabolomic parameters. The collection of previous studies that analyzed IGF pathway molecules as potential biomarkers for risk of development of disease, unfortunately, has neither been able to conclusively describe the definitive actions of such molecules, nor resolve the significance of the pathway with respect to the disease onset or progression. Despite the acknowledged limitations, the inclusion and combination of members from the IGF axis in panels of biomarkers and with LDCT scans have strengthened the efficacy of lung cancer detection methodologies and show great promise for inclusion in biomarker panels aimed at improved clinical decision making. Nevertheless, further research with a focus on a wider range of molecules within the IGF system and larger sample sizes are required to confirm these results. Until the coordinated integral standardization of assay protocols has been implemented, refined, and incorporated into large-scale and generalizable studies, the current data is merely a source of speculative guidance regarding real-world treatment tactics and strategies. If such procedural advancements do occur, the realization of IGF biomarkers as potential ambassadors of therapy or agents of surveillance against and of the disease could radically alter the landscape of lung cancer diagnostics, prognostics, and treatment. Such aspirations can only be achieved with continued federal funding to support further research, development, and implementation of these systems into lung cancer detection and treatment modalities.
